# ONP-302 Nanoparticles Inhibit Tumor Growth By Altering Tumor-Associated Macrophages And Cancer-Associated Fibroblasts

**DOI:** 10.7150/jca.69338

**Published:** 2022-03-28

**Authors:** Laxminarasimha Donthireddy, Prashanthi Vonteddu, Tushar Murthy, Taekyoung Kwak, Rukiye-Nazan Eraslan, Joseph R. Podojil, Adam Elhofy, Michael T. Boyne, John J. Puisis, Filippo Veglia, Surya S. Singh, Farokh Dotiwala, Luis J. Montaner, Dmitry I. Gabrilovich

**Affiliations:** 1 Immunology, Microenvironment and Metastasis Program, The Wistar Institute, Philadelphia, PA, 19104, USA.; 2 Vaccine and Immunotherapy Center, The Wistar Institute, Philadelphia, PA, 19104, USA.; 3 Research & Development, Cour Pharmaceuticals Development Company, Northbrook, IL, USA.; 4 Invivotek, Genesis Drug Discovery and Development (GD3), Hamilton, NJ, USA.; 5 Department of Microbiology and Immunology, Northwestern University, Chicago, IL, USA.; 6 Current affiliation: H. Lee Moffitt Cancer Center, Tampa, FL.; 7 Department of Biochemistry, Osmania University, Hyderabad, India.; 8 Current affiliation: ICC, Early Oncology R&D, AstraZeneca, Gaithersburg, 20878, USA.

**Keywords:** MDSC, Tumor Microenvironment, Nanoparticles, Tumor Associated Macrophages (TAMs), Cancer Associated Fibroblasts (CAFs), PMN-MDSC, M-MDSC.

## Abstract

In this study, we evaluated the ability of negatively charged bio-degradable nanoparticles, ONP- 302, to inhibit tumor growth. Therapeutic treatment with ONP-302 in vivo resulted in a marked delay in tumor growth in three different syngeneic tumor models in immunocompetent mice. ONP- 302 efficacy persisted with depletion of CD8+ T cells in immunocompetent mice and also was effective in immune deficient mice. Examination of ONP-302 effects on components of the tumor microenvironment (TME) were explored. ONP-302 treatment caused a gene expression shift in TAMs toward the pro-inflammatory M1 type and substantially inhibited the expression of genes associated with the pro-tumorigenic function of CAFs. ONP-302 also induced apoptosis in CAFs in the TME. Together, these data support further development of ONP-302 as a novel first-in- class anti-cancer therapeutic that can be used as a single-agent as well as in combination therapies for the treatment of solid tumors due to its ability to modulate the TME.

## Background

The tumor microenvironment (TME) plays a crucial role in tumor growth and progression and has a significant influence on response to therapy 1,2. The TME consists of myeloid-derived cells, stroma (e.g. fibroblasts and extracellular matrix (ECM)), and the vasculature that together support tumor growth and progression. Studies in animal models and in humans show that myeloid- derived cells such as myeloid derived suppressor cells (MDSCs) and tumor associated macrophages (TAMs) engage in activities that support tumor growth and progression 3,4. These cells also promote immune suppression in the TME that blunts the efficacy of the anti-cancer drugs 5,6 and immune-targeted therapies such as immune checkpoint inhibitors 7. TAMs are a phenotypically plastic cell type existing within the TME, and these cells can functionally differentiate between either M1 pro-inflammatory (CD86+ iNOS+ HLA-DR+) or M2 anti-inflammatory (CD206+ Arg+) phenotypes 8,9. The signaling milieu of the TME is skewed towards promoting M2 type TAM with immune-suppressive and pro-tumor functions 10. MDSCs can be classified into two major sub-types based on morphology and cell-surface marker expression: monocytic myeloid-derived suppressor cells (M-MDSCs) (CD11b+ Ly6Chi Ly6G-) and polymorphonuclear cells myeloid-derived suppressor cells (PMN-MDSCs) (CD11b+ Ly6C- Ly6G+). M-MDSCs can rapidly differentiate into TAMs in response to signaling molecules present at the TME. Thus, M-MDSCs and TAMs represent different stages of differentiation of the same cell-lineage 11-13. The presence of MDSCs in peripheral blood and TAMs in the TME predicts poor survival and is associated with low rates of response and development of resistance to immune-targeted therapies such as immune checkpoint inhibitors 7,14-18.

In addition to myeloid-derived immune cells, fibroblasts are one of the more abundant cell-types found in the TME 19. Fibroblasts are stromal cells that perform several essential functions that are important in providing mechanical support to the neighboring epithelium, tissue organization and structure, remodeling of the ECM, production of growth-factors, and wound healing 20. Fibroblasts are generally quiescent under normal physiological conditions but are activated during tumorigenesis via complex signaling pathways to support tumor growth and progression 21,22. Fibroblasts phenotypically change within the tumor during disease progression and undergo significant changes in their proliferative capacity, migratory potential, and gene expression patterns that result in phenotypic and functional shifts that support tumor growth. Such activated fibroblasts in the TME are termed cancer-associated fibroblasts (CAFs) 23,24. CAFs support tumor progression via production of pro-tumor and pro-angiogenic growth-factors, remodeling of the ECM via production of proteases, and suppression of anti-tumor immune function 25-27. Furthermore, CAFs contribute to fibrosis in the TME which acts as a physical barrier preventing immune-cell infiltration into the TME and negatively interferes with the distribution of anti-cancer therapeutics 28,29. CAF abundance in the TME is a negative prognostic factor for several solid tumors and is associated with negative outcomes and poor response to immune-targeted therapies like immune checkpoint inhibitors 27,30.

Here, we examined ONP-302 nanoparticles, previously described in the literature as Immune Modifying Particles (IMPs/PLG-IMP/PS-IMP) 31, for the treatment of cancers. ONP-302 particles are fabricated from biodegradable poly (lactic-co-glycolic acid)(PLGA) polymer and are free from encapsulated or attached drugs. The physiochemical properties of ONP-302 particles are designed specifically for targeted uptake and modulation of myeloid-derived cells such as monocytes, macrophages, and neutrophils via the scavenger receptor MARCO 31. ONP-302 is composed of PLGA polymer nanoparticles 400-800 nm in size and a negatively charged surface with a zeta potential between -35 mV and -50 mV.

Tumor immune cell infiltrate and the functional phenotype of these infiltrating cells is a major determinant of tumor response to immunotherapies (e.g., checkpoint inhibitors). High levels of tumor infiltrating cytotoxic T cells are associated with strong responses to checkpoint inhibitors (e.g., anti-PD1) 32. In contrast, low levels of tumor infiltrating cytotoxic T cells and/or high levels MDSCs and M2 TAMs are associated with poor response to immunotherapies inhibitors, treatment resistance, and poor outcomes 15,33,34. Previous studies in several pre-clinical models of acute inflammation have demonstrated that ONP-302 treatment resolves inflammation via targeted inhibition of pro- inflammatory Ly6Chigh monocyte, macrophage, and neutrophil trafficking into sites of active inflammation 31,35-38. Since MDSCs and TAMs are derived from the same myeloid cell populations cells targeted by ONP-302, we hypothesized that ONP-302 treatment could prevent MDSC and TAM infiltration and accumulation within the TME, disrupt tumor-promoting and immunosuppressive signaling pathways, and allow tumor growth control via subsequent activation of effector immune cell (T cells and NK cells) function. Along this same line of reasoning, we further hypothesized that treatment with ONP-302 might enhance the efficacy of immune checkpoint inhibitors. In addition to exploring the immunomodulatory anti-tumor effects of ONP- 302, we examined effects on pro-tumorigenic cells in the TME.

We report here that therapeutic treatment with ONP-302 was highly effective at decreasing/slowing tumor growth in several murine syngeneic tumor models. We found ONP-302- positive TAMs and CAFs in the TME and treatment with ONP-302 resulted in a gene expression shift in TAMs from M2 to M1 type along with inhibition of pro-tumorigenic gene-expression in CAFs within tumors. Additionally, particle uptake was associated with the induction of apoptosis in CAFs in the TME. Together, these findings indicate a mechanism whereby ONP-302 treatment slows tumor growth by altering pro-tumorigenic TAMs and CAFs in the TME.

## Materials and Methods

**Mice:** All animal procedures were approved by the Wistar institutional animal care and use committee. 6-week-old female C57BL/6 mice were purchased from the Charles River. NOD- SCIDγ (NSG) mice were bred in the Wistar facility. All the mice were maintained in pathogen- free temperature-controlled room 12/12hr dark/light mode and food provided ad libitum. Mice were randomized to different groups based on equal tumor size (~50 mm^2^) before the start of therapeutic experiments.

**ONP-302:** ONP-302 immune modifying nanoparticles were manufactured by COUR Pharmaceuticals. Particles were made from poly (lactic-co-glycolic acid) (PLGA) (Lactel®, Durect Corporation) using a double emulsion technique using a proprietary blend of solvents, surfactants, and stabilizers. ONP-302 particles have an average diameter of 500 nm and a zeta potential of approximately -40mV. Particles were resuspended in 0.9% saline for the *in vivo* studies.

**ONP-302 particle uptake:** LLC tumor-bearing mice were injected with 50 mg/kg ONP-302 labeled with OVA-AlexaFluor-647 via intravenous injection. 2 hours after injection, mice were euthanized. Tumors and spleens were harvested, and single-cell suspensions were prepared. Cells were stained with indicated antibodies listed in **Supplementary Table 1** and analyzed on a BD LSRII flow cytometer. Data analysis was performed using FlowJo Software (Tree Star).

**Reagents and cell lines:** Tumor cell lines LLC (Lewis Lung Cancer), MC-38 (Colon Carcinoma), and B16.F10 (Melanoma) were obtained from ATCC (46). All cells were maintained in DMEM medium supplemented with 10% fetal bovine serum (FBS; Sigma-Aldrich) at 37 °C, 5% CO2. Tumor cell-lines obtained from ATCC were tested for mycoplasma contamination by using the Universal Mycoplasma Detection kit (ATCC) every 3 months.

**Tumor cell injections and treatment:** Approximately 0.5 × 10^6^ LLC, 1 × 10^6^ MC38, or 0.2 × 10^6^ B16F10 tumor cells were injected into shaved flanks of mice via subcutaneous injection. ONP-302 was administrated intravenously at a dose of 50 mg/kg twice a week after palpable tumor formation (~50 mm^2^). Saline i.v injection was used as control. The tumor size was measured by vernier calipers before and after the treatment day of every ONP-302 injection. Anti-PD-1 antibody (clone RMP1-14, BioXcell, 5 mg/kg (LLC, MC-38) or 10 mg/kg (B16F10)) was administered two times per week via intraperitoneal injection after palpable tumor formation. Tumor sizes were calculated using the formula: Tumor size (mm^2^) = length × width.

***In vitro* tumor cell-viability assay:** 2 × 10^3^ tumor cells (LLC and MC-38) were cultured in 100 μl of RPMI complete media in a 96-well plate and incubated for 24 hours at 37 °C, 5% CO2. ONP-302 was added to each well at the indicated concentrations. 72 hours after incubation, 20 μL of the resazurin dye (already in solution at a fixed concentration as prepared by the manufacturer (Sigma)) was added to each well. The plate was placed onto an orbital shaker for 5 minutes. The plate was then incubated at 37 °C, 5% CO2 for 2-4 hours. Absorbance was measured at 600 nm. **RT-qPCR:** RNA was extracted from sorted cells using the Total RNA Kit according to the manufacturer's instructions (Zymo Research kit). cDNA was generated using High- Capacity cDNA Reverse Transcription Kit (Applied Biosystems). RT-qPCR was performed with primers listed in **Supplementary Table 2** using Power SYBR Green PCR Master Mix (Applied Biosystems) in 96 well plates. Primer had an annealing temperature of 60 °C and elongation temperature of 70°C. Plates were read with ABI 7500 fast RT PCR (Applied Biosystems). Relative gene expression was normalized to GAPDH and calculated by the comparative C(T) method 42.

**Preparation of single-cell suspensions from mouse tissues and staining:** Spleens and tumors were harvested from mice and homogenized by using shear force. Tumor single-cell suspensions were prepared using Mouse Tumor Dissociation Kit according to the manufacturer's recommendation (Miltenyi Biotech). Red blood-cell lysis was performed using ACK lysis buffer. Antibodies specific for the mouse cell surface markers CD11b, Ly6C, Ly6G, CD45, CD140a (PDGFRa), ANNEXIN V and F4/80 were used and are listed in **Supplementary Table 1**. The flow cytometry data were acquired using BD LSRII flow cytometer and data analysis was performed using FlowJo Software (Tree Star).

**Masson's Trichrome staining:** Deparaffinize the sections and rehydrate through 100% alcohol, 95% alcohol and 70% alcohol. Wash them in distilled water before fix in Bouin's solution (Poly Scientific, Cat # S129) for 1 hour at 56 C. Then rinse under the running tap water for 5-10 minutes and stain in Weigert's iron hematoxylin (ScyTek, Cat # HWI-A-125) for 15 minutes. Then stain in Biebrich scarlet-acid fuchsin solution (Poly Scientific, Cat # S125) for 3 minutes after washing them under running warm tap water and distilled water for 10 minutes. Wash and differentiate in phosphomolybdic-phosphotungstic acid solution (Poly Scientific, Cat # S255) for 25 minutes. Transfer the sections to aniline blue solution (ScyTek, Cat # ABP125) for 15 minutes. Then wash and dehydrate very quickly through 95% alcohol, 100% alcohol, and in xylene. Then mount with mounting medium.

**Immunofluorescence staining:** Frozen tissue sections were air-dried for 10 minutes and fixed in Methanol at -20° for 10 minutes. Sections were washed in DI water and treated with TX-100 in PBS for 15 minutes. Then washed them with PBST and add 2.5% Horse Serum for 1 hr and primary antibody Anti-Actin α Smooth Muscle antibody (Sigma, cat# A5228) is applied to the sections for overnight incubation at 4°. Then sections were washed with PBST and stained with secondary antibody (Invitrogen, Cat# A27023**)** applied 1:200 dilution for 30 minutes at room temp. Quenching solution were applied for 3 minutes before washing with PBST. Then wash them and add DAPI for 5 minutes. And rinse in DI water and coverslip with water-based mounting medium. Specimens were documented photographically using Leica TCS SP5 Scanning Confocal Microscope.

**Statistical analysis:** Statistical analyses were performed using GraphPad Prism 5 software (GraphPad Software Inc.), two-tailed Student t test or Mann-Whitney U test and Paired t-test were used since data were normally distributed. Statistical differences in tumor growth were evaluated using Two-Way Anova test with correction for multiple measurements. All the data are presented as mean ± SD and P value < 0.05 was considered statistically significant.

## Results

ONP-302 nanoparticles were manufactured using a double-emulsion technique and a proprietary blend of surfactants and stabilizers optimized to yield nanoparticles in the desired size (400-800 nm) and zeta potential (-35 to -50 mV). Particles had a diameter of 568 ± 13 nm and a zeta potential of -41.9 ± 0.03 mV **(Figure S1, Supplementary Table 1),** optimized for receptor-mediated phagocytic uptake by myeloid-derived cells. We tested the efficacy of ONP-302 in three different syngeneic murine tumor models: B16.F10 melanoma (B16), Lewis Lung Carcinoma (LLC), and MC-38 colon carcinoma that differ fundamentally in several aspects including growth characteristics, immune composition, and responsiveness to immune checkpoint inhibitors. B16 tumors are considered 'cold tumors' due to low immunogenicity, low levels of tumor immune infiltrate, and resistance to immunotherapies such as checkpoint inhibitors (e.g., anti-PD1). LLC tumors are moderately immunogenic exhibiting intermediate levels of tumor immune infiltrate and modest response to checkpoint inhibitors. MC- 38 tumors, in contrast, are considered 'hot tumors' due to high immunogenicity, high levels of tumor immune infiltrate, and strong response to checkpoint inhibitor therapy 39. These tumor cell lines were selected for the study of ONP-302 anti-tumor efficacy to account for heterogeneity of human tumors and to determine whether tumor characteristics influenced efficacy.

**Figure 1:**
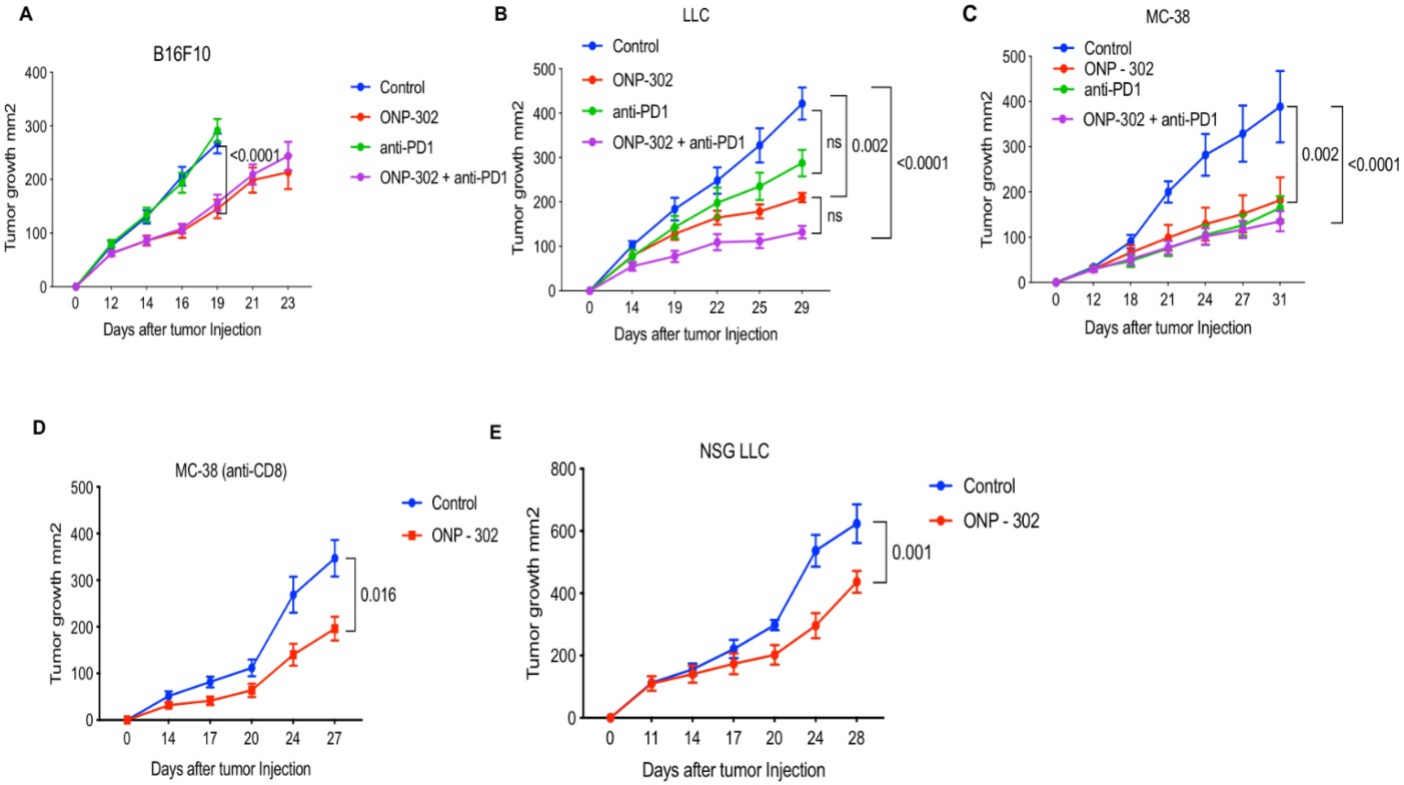
** Effect of ONP-302 on tumor growth in different tumor models.** C57BL/6 mice (n=10) were injected with 1 x 10^6^ MC-38 cells and 0.5 x 10^6^ LLC and B16F10 cells. Treatments with ONP-302 intravenously 50mg/kg biw started at ~50 mm^2^ tumor size after tumor injection. Anti-PD1 antibody (100μg per mouse, intraperitonially) administered biw. Tumor growth of **(A)** B16 **(B)** LLC and **(C)** MC-38 tumor bearing mice administered the indicated treatments. **D**. Tumor growth in MC-38 tumor-bearing C57BL/6 mice administered anti-CD8 for T-cell depletion and treated with ONP-302 or Saline (Control). **E**. Tumor growth in LLC tumor-bearing NSG mice treated with ONP-302 or Saline (Control). The tumor size was measured by vernier calipers before and after the treatment day of every ONP-302 injection. Statistical differences in tumor growth were evaluated using Two-Way Anova test (***p<0.001).

In all three models, treatment with ONP-302 alone led to a significantly reduced tumor burden. In the anti-PD1 resistant B16 tumor model, ONP-302 treatment led to significantly reduced tumor burden (45% reduction) (p<0.0001) compared to Control treatment on Day 16 **(Figure 1A).** Tumor growth assessments in the Control group were not possible after Day 16 as a significant number of animals in this group had to be euthanized for humane reasons due to a potentially terminal condition and/or large tumor sizes when compared to the ONP-302 treated groups where tumor growth was monitored up to Day 23. As expected, anti-PD1 checkpoint inhibitor treatment had no effect on B16 tumor growth and tumor burden at Day 16 was comparable to Control treatment. Combination therapy with ONP- 302 and anti-PD1 led to 41% reduction in tumor burden compared to Control treatment on Day 16. Treatment with anti-PD1 antibody did not enhance the effect of ONP-302 therapy **(Figure 1A).** In the moderately immunogenic LLC tumor model **(Figure 1B),** ONP-302 treatment led to a 64% reduction in tumor growth compared to Control (p=0.002). In comparison, anti-PD1 treatment demonstrated a modest 43% reduction in tumor burden which did not reach statistical significance. Remarkably, treatment with ONP-302 and anti-PD1 in combination led to a statistically significant 72% reduction in tumor burden (p<0.0001) compared to control. However, the differences between ONP-302 treated group and combination group did not reach statistical significance. Finally, in the anti-PD1 responsive MC-38 model **(Figure 1C),** ONP-302 treatment led to a 47% reduction in tumor burden compared to control group (p=0.002). ONP-302 was as effective as anti-PD1 - a significant finding given how responsive MC-38 tumors are to checkpoint blockade. Combination therapy with ONP-302 and anti-PD1 led to a 61% reduction in tumor burden compared to the control treatment group (p<0.0001), which was not statistically different when compared to respective monotherapies. In summary, robust ONP-302 efficacy against three distinct tumors with varying degrees of immunogenicity and tumor immune infiltrate suggested that a putative effect on tumor growth may be due to a common mechanism, independent of tumor- specific factors. Treatment of mice with ONP-302 in combination with check-point inhibitor did not lead to a synergistic effect on tumor growth inhibition suggesting that ONP-302 efficacy may not be entirely mediated by effector immune function.

To determine whether the efficacy of ONP-302 was due to a direct tumoricidal property, we incubated ONP-302 with tumor cells in vitro. We did not observe any evidence of tumor cell-death **(Figure S2),** which suggests that ONP-302-induced decrease in tumor growth in vivo was unlikely due to a direct tumor killing function of ONP-302.

Next, we examined whether ONP-302 efficacy was via induction of a T cell mediated anti-tumor immune response. We examined ONP-302 efficacy after anti-CD8 antibody-mediated CD8+ T cell depletion in wild-type C57BL/6 mice bearing MC-38 tumors. Despite depletion of CD8+ T cells, ONP-302 treatment led to a statistically significant 47% reduction in tumor burden (p=0.016) **(Figure 1D).** In another experiment, we used immunodeficient NOD-SCIDγ (NSG) mice which lack effector immune function. ONP-302 reduced tumor burden in LLC tumor-bearing NSG mice by 30% compared to control treatment group (p=0.001) **(Figure 1E).** These data indicated that the antitumor effect of ONP-302 treatment may not depend solely on T cells.

**Figure 2:**
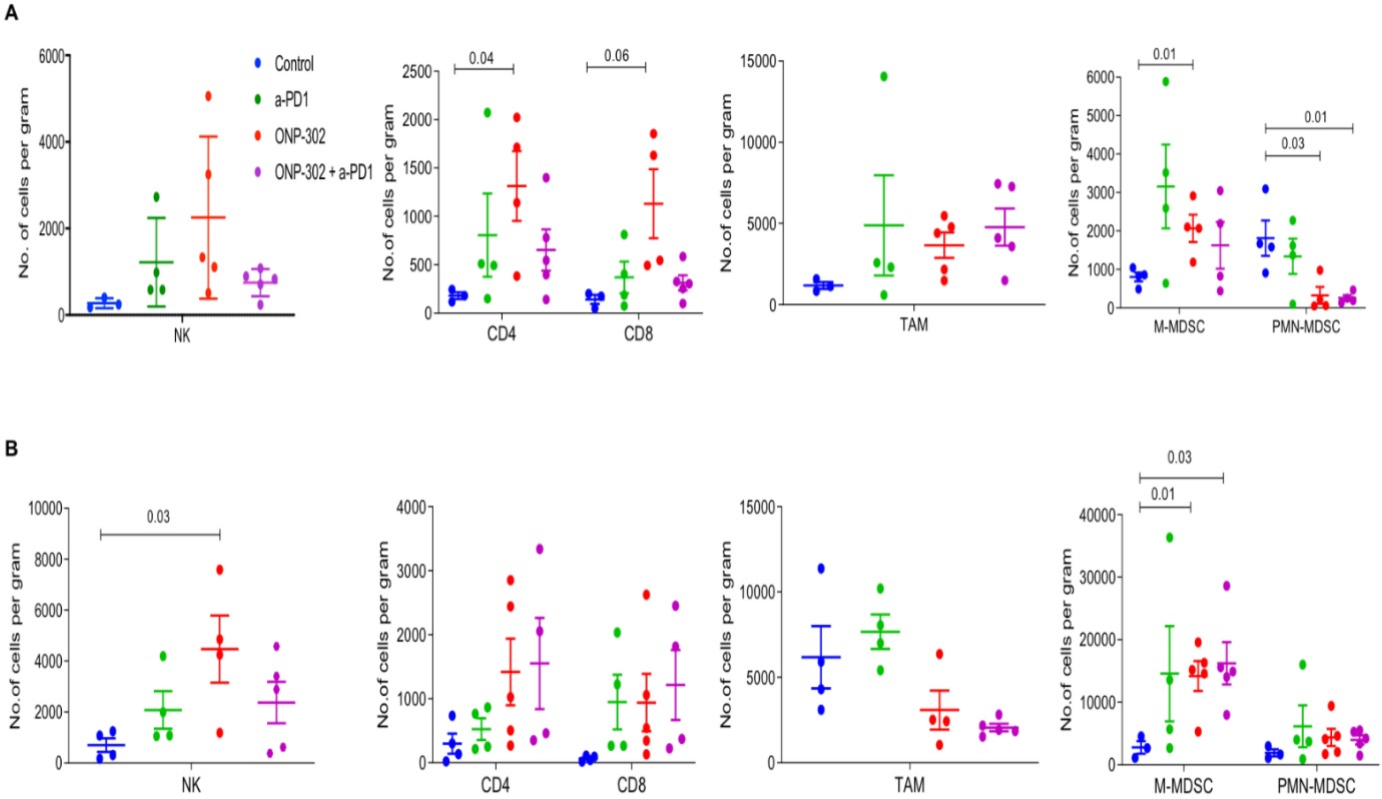
** Effect of ONP-302 on immune-cell composition in the TME**. LLC (**A**) tumor bearing C57BL/6 mice and MC-38 (**B**) tumor-bearing C57BL/6 mice were treated with vehicle or ONP-302 50mg/kg intravenously twice per week for 2 weeks. Single-cell suspensions were prepared from tumor and analyzed by flowcytometry. Percentages of T-cells (CD3+CD4+ or CD3+CD8+), NK cells (CD3- NK1.1+), TAM (CD11b+F4/80+), M-MDSC (CD11b+ Ly6Chi Ly6G-), and PMN-MDSC (CD11b+ Ly6C- Ly6G+) were measured.

Previously published data showed that ONP-302 particles targeted monocytes, macrophages, and neutrophils 31. We examined the effect of ONP-302 treatment on myeloid and lymphoid immune cell populations from tumors and spleens of LLC and MC-38 tumor-bearing mice using flow cytometry. To account for the differences in tumor sizes in the ONP-302 and Saline (Control) treatment groups, we measured changes in these cell populations per gram of the tumor harvested for analyses. ONP-302 treatment had no effect on the number of TAMs (CD11b+F4/80+) in both LLC and MC-38 tumors; however, we observed a statistically significant increase in the number of M-MDSCs (CD11b+/Ly6Chi/Ly6G-) per gram of tumor after ONP-302 treatment in both LLC (p=0.01) and MC-38 tumors (p=0.01). PMN-MDSCs (CD11b+/Ly6C-/Ly6G+) numbers, in contrast, were reduced significantly in LLC tumors (p=0.03) but not in MC-38 tumors **(Figures 2A and 2B)**. Examination of T cells (CD3+/CD4+ and CD3+/CD8+) and NK cells (CD3-/NK1.1+), primarily responsible for anti-tumor effector immune function, revealed statistically significant increases in the numbers of CD4+ T cells (p=0.04) in LLC tumors **(Figure 2A)** and NK cells in MC-38 tumors (p=0.03) **(Figure 2B).** We did not find evidence of changes in myeloid or lymphoid cell percentages in the spleen (Figure S3). Combination therapy with ONP-302 and anti-PD1 also did not appear to alter myeloid and lymphoid cell numbers in tumors and spleens to biologically meaningful degrees **(Figure 2 and Figure S3)**. Thus, ONP-302 treatment did appear to alter some myeloid and lymphoid cell types in the TME, but these changes were inconsistent between tumor models and suggested that an alternative mechanism of action may also exist.

**Figure 3:**
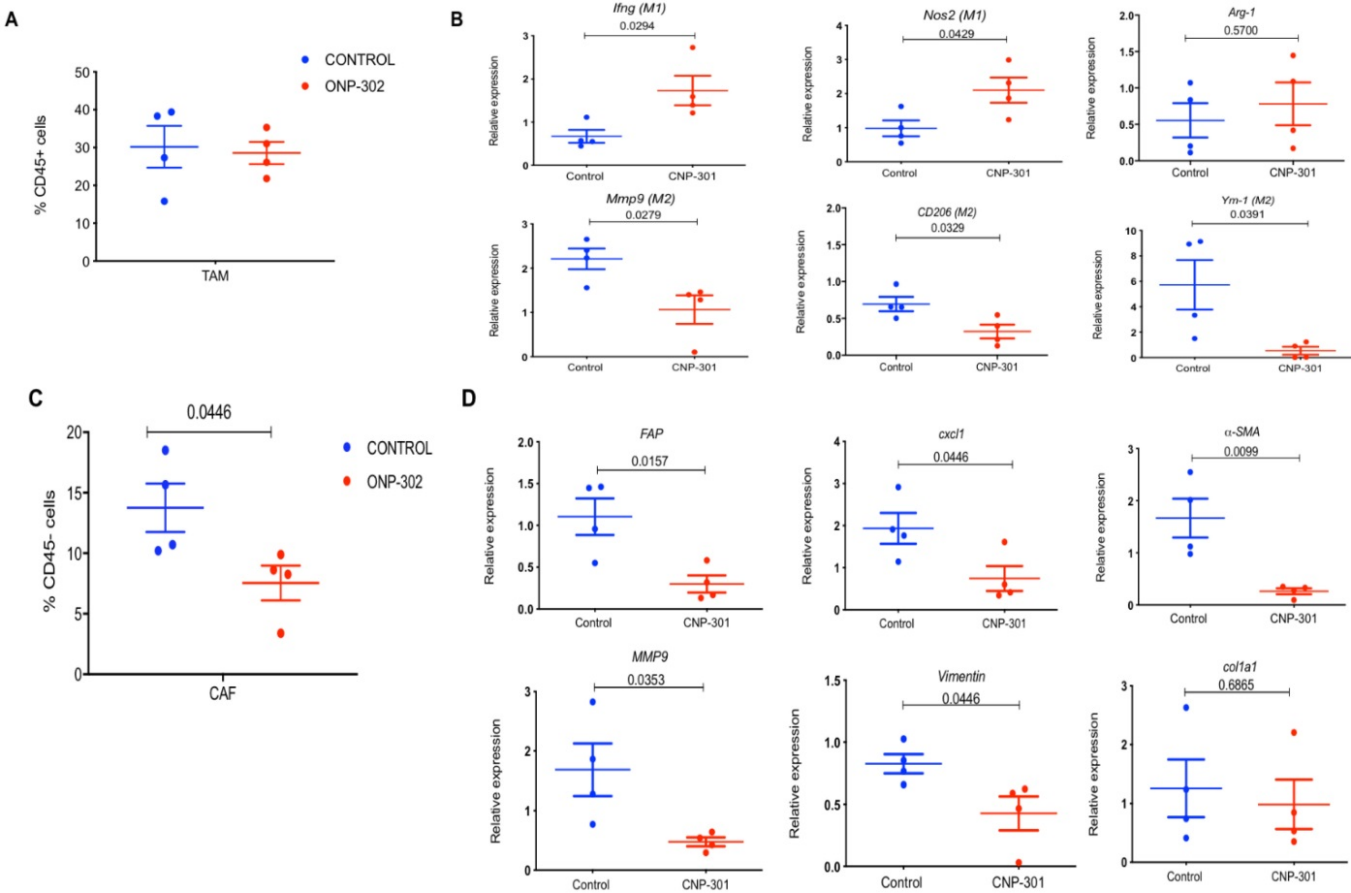
**Effect of ONP-302 on gene expression in tumor-associated macrophages (TAMs) and fibroblasts.** LLC tumor bearing C57BL/6 mice were treated with vehicle or ONP-302 50mg/kg intravenously twice per week for 2 weeks. Single-cell suspensions were prepared from tumor. TAMs and fibroblasts were sorted by fluorescence activated cell-sorting. **A**. Effect of ONP-302 treatment on the percentage of TAM (CD11b+F4/80+) in the tumor. **B**. Effect of ONP-302 on the expression of indicated genes in TAMs assayed by q-PCR. Relative gene expression was normalized to GAPDH. **C.** Effect of ONP- 302 treatment on the percentage of CAFs (CD45-CD140a+CD326-) in the tumor. Effect of ONP-302 on the expression of indicated genes in CAFs assayed by q-PCR. Relative gene expression was normalized to GAPDH and calculated by the comparative C(T) method 42.

Next, we examined the possibility that ONP-302 could induce functional changes in TAMs. We sorted TAMs (CD11b+F4/80+) from LLC tumors after control or ONP-302 treatment and examined gene expression patterns associated with the pro-inflammatory/anti-tumor M1 and anti- inflammatory/pro-tumor M2 types of TAMs. Consistent with earlier observations, ONP-302 did not alter the total number of TAMs in LLC tumors **(Fig. 3A);** however, there was a clear shift in gene expression from an anti-inflammatory/pro-tumor M2 to a pro-inflammatory M1 state of TAM. We found significantly increased expression of *Ifnγ* (p=0.0294) and *Nos2* (p=0.0429) genes associated with M1 TAMs, and significantly decreased expression of *CD206* (p=0.0329) and *Ym1* (p=0.0391) genes associated with M2 TAMs after ONP-302 treatment **(Figure 3B).** Additionally, expression of *Mmp9* encoding for an ECM remodeling protease implicated in tumor progression and metastasis also was decreased significantly (p=0.0279) **(Figure 3B).**

We then examined whether ONP-302 could directly alter the polarization of macrophages. Treatment of macrophages generated from bone marrow monocytes from naïve mice with ONP-302 *in vitro* polarized them towards M1 type **(Figure S4).**

Presence of TAMs and CAFs in close vicinity within the TME and an interplay between these cells via chemokine signaling pathways has been reported 40,41. In light of our observations of gene expression changes in TAMs after ONP-302 treatment, we examined whether CAFs were also affected. Expression of several genes associated with CAFs and their pro-tumorigenic activity were examined. ONP-302 treatment reduced the total number of CAFs (CD45-CD140a+CD326-) in tumor tissues **(Figure 3C)** and led to a statistically significant reduction in the expression of *Fap* (p=0.0157), *Cxcl1* (p=0.0446), *αSma* (p=0.0099), and *Vim* (p=0.0446) mRNA associated with ECM remodeling and pro-tumorigenic function **(Figure 3D).** Like TAMs, *Mmp9* expression was significantly decreased in fibroblasts isolated from LLC tumors after ONP-302 treatment (p=0.0353) **(Figure 3D).** These results demonstrate that ONP-302 treatment resulted in gene expression changes in two major cell-types in the TME indicative of disruption of the pro-tumor TME.

**Figure 4:**
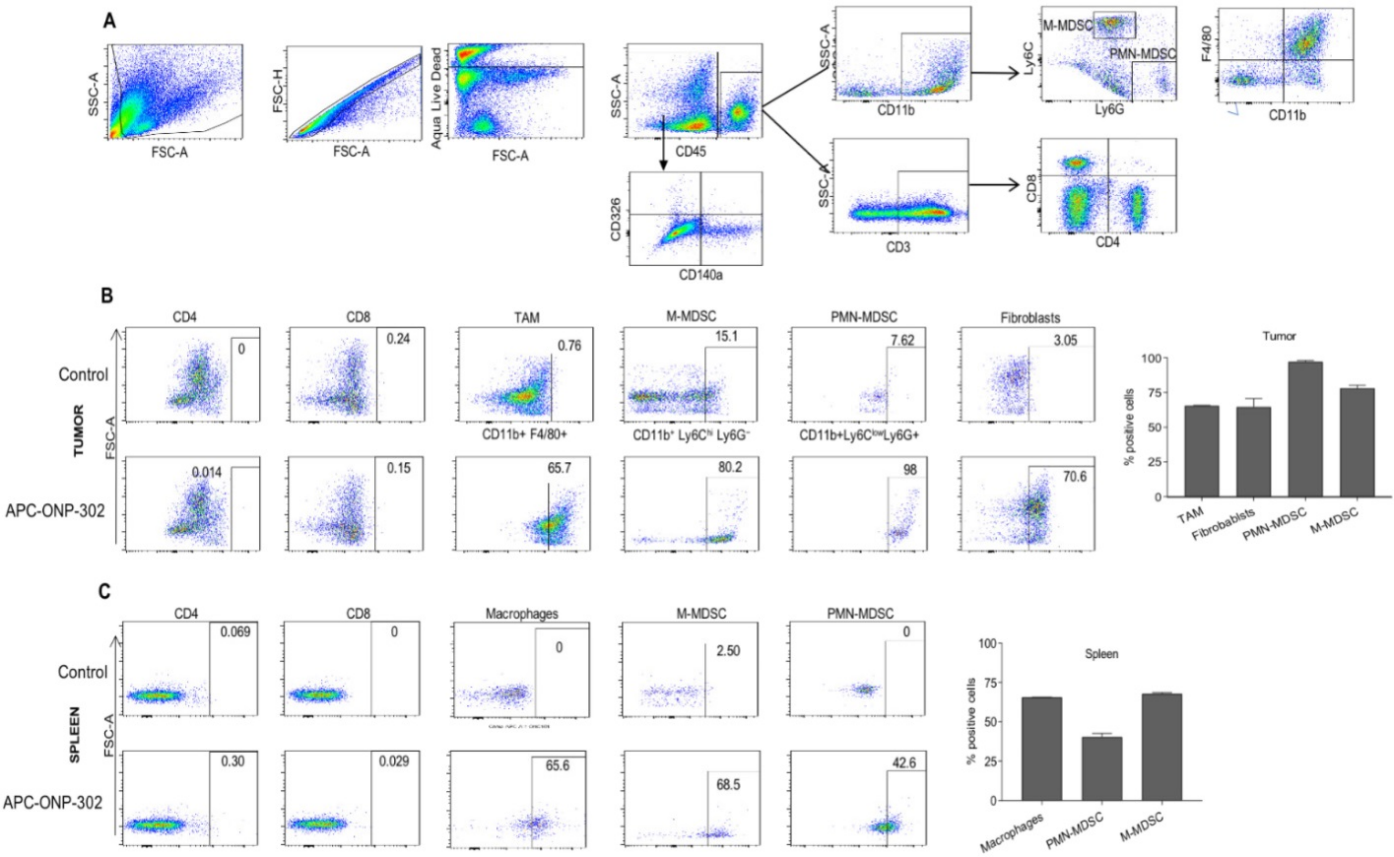
** Evaluation of particle-positive cells in the tumor and spleen**. LLC tumor bearing C57BL/6 mice were intravenously treated injected with fluorescently labelled (Alexa-Fluor 647) ONP-302 50mg/kg. 2 hours after injection, single-cell suspensions were prepared from tumor and spleen and ONP-302-positive cells were analyzed by flow cytometry. **A**. Flow cytometry gating scheme for identifying ONP-302-positive TAMs (CD11b+F4/80+), M-MDSC (CD11b+ Ly6Chi Ly6G-), and PMN-MDSC (CD11b+ Ly6C- Ly6G+), and CAFs (CD45-CD140a+CD326-). **B**. Frequency of Alexa-Fluor 647-positive TAMs (CD11b+F4/80+), M-MDSC (CD11b+ Ly6Chi Ly6G-), and PMN-MDSC (CD11b+ Ly6C- Ly6G+), and CAFs (CD45- CD140a+CD326-) in LLC tumors. **C**. Frequency of Alexa-Fluor 647-positive macrophages (CD11b+F4/80+), monocytes (M-MDSC) (CD11b+ Ly6Chi Ly6G-), and neutrophils (PMN-MDSC) (CD11b+ Ly6C- Ly6G+) in spleens of LLC tumor-bearing mice.

To address the question of whether gene expression changes in TAMs and CAFs in the TME were a result of ONP-302 uptake, we injected fluorescently labeled ONP-302 particles into LLC tumor- bearing mice and assessed ONP-302-positive cells by flow cytometry **(Figure 4A).** Consistent with previously reported data showing myeloid uptake of ONP-302, a majority of TAMs (65.7%), M-MDSCs (80.2%), and PMN-MDSCs (98%) in LLC tumors were positive for ONP-302 at 2-hours after injection **(Figure 4B).**

Very few ONP-302-positive cells were observed at later timepoints (data not shown). In addition to myeloid-derived cells, 75% CAFs in LLC tumors were also ONP- 302 positive at the 2-hour timepoint **(Figure 4B).**

Examination of myeloid cells in the spleen also revealed particle positive macrophages (65.6%), monocytes (M-MDSC) (68.5%), and neutrophils (PMN-MDSC) (42.6%). Of note, fewer neutrophils in spleen were ONP-302-positive when compared to the tumor (42.6% vs. 98%) (**Figures 4B and 4C).** We found no evidence of ONP-302-positive T cells in the tumor or spleen. These data provided evidence that gene expression changes in TAMs and CAFs were a direct result of ONP-302 treatment.

Since PMN-MDSC were able to pick-up ONP-302, we asked if this uptake resulted in changes in their ability to suppress T cells. PMN-MDSC were sorted from tumors of untreated or ONP-302 treated LLC tumor-bearing mice and their ability to suppress antigen-specific T-cell proliferation was assessed using an *in vitro* co-culture assay. Proliferation of T-cells was measured by assaying tritiated thymidine (^3^H) incorporation. PMN-MDSC from untreated mice demonstrated potent suppressive activity. Suppressive activity of PMN-MDSC was substantially reduced in mice treated with ONP-302 as indicated by reduced ^3^H incorporation in T cells** (Figure S5).**

**Figure 5:**
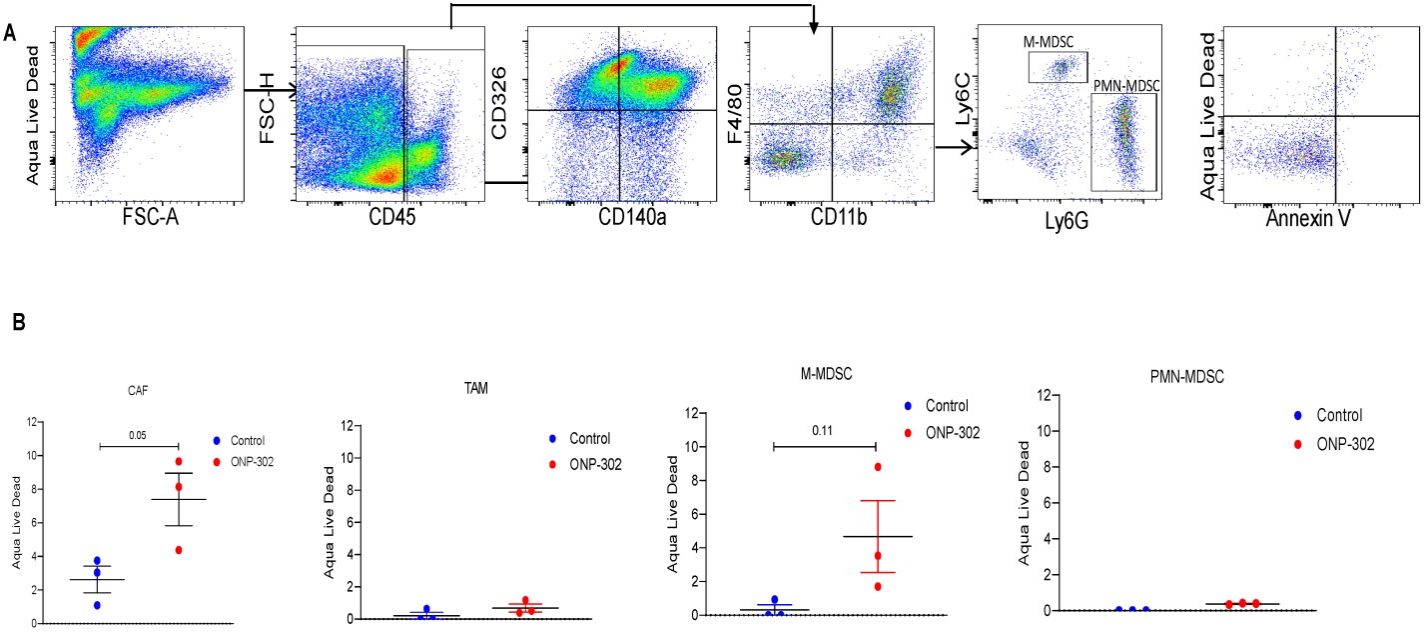
** Induction of Apoptosis by ONP-302.** LLC tumor bearing C57BL/6 mice were intravenously injected with fluorescently labeled (Alexa-Fluor 647) ONP-302 50mg/kg. for 2 weeks. 2 hours after last treatment, mice were sacrificed, and single-cell suspensions were prepared from the tumor and spleen. Induction of Apoptosis by ONP-302 was analyzed by flow cytometry. **A**. Flow cytometry gating scheme for identifying apoptotic TAMs (CD11b+F4/80+), M-MDSC (CD11b+ Ly6Chi Ly6G-), and PMN-MDSC (CD11b+ Ly6C- Ly6G+) and CAFs (CD45-CD140a+CD326-) using the indicated cell-surface markers. **B**. Flow cytometry analysis of indicated apoptotic cells in tumor.

In light of evidence of ONP-302 uptake by CAFs in the tumor and reduction in their overall numbers in the tumor after ONP-302 treatment, we examined whether particle uptake induced apoptosis in CAFs or other cells. Flow cytometric evaluation of apoptosis in tumor associated cells **(Figure 5A)** showed an increase in apoptotic CAF but not TAM or PMN-MDSC in ONP-302 treated mice as compared to control **(Figure 5B).** There was a trend in increase in cell death in M- MDSC after the treatment with ONP-302. However, it did not reach statistical significance.

**Figure 6:**
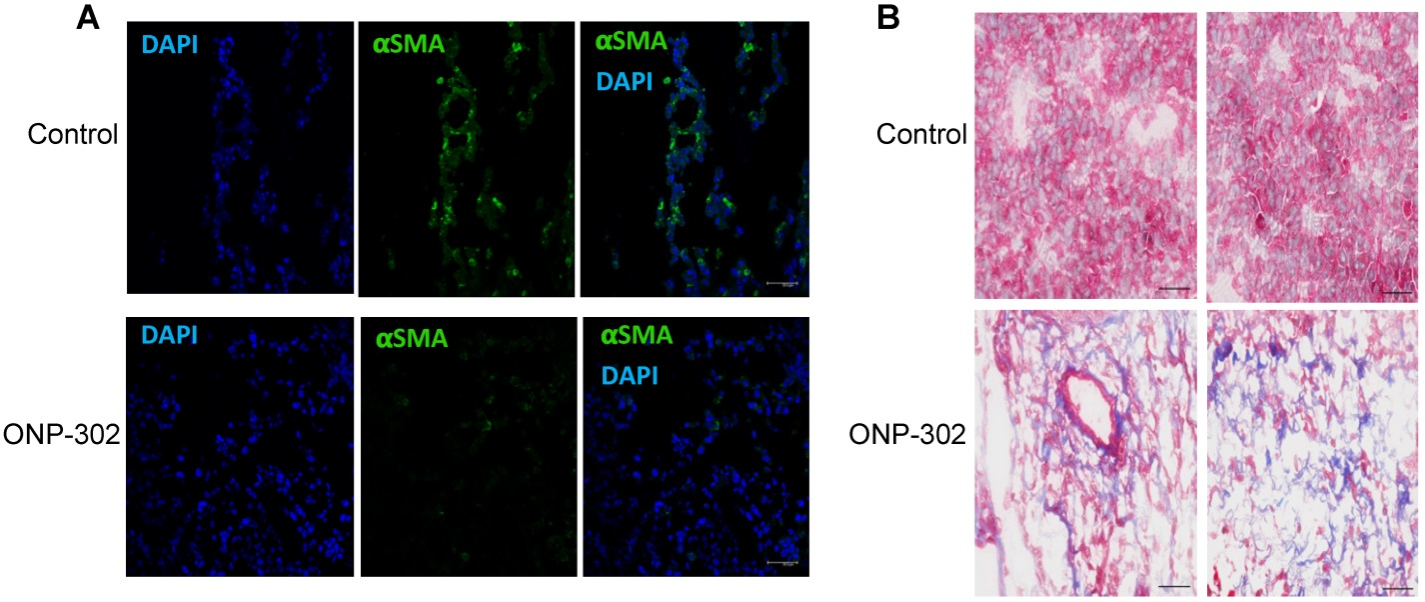
Apoptosis of cancer associated fibroblasts (CSF) in LLC mice treated with CNP-302. LLC tumor-bearing mice were treated with ONP-302 (50mg/kg) intravenously twice per week for 2 weeks and tumor tissues were prepared for the staining. **A.** Typical example of tumor tissue staining with αSMA. Scale = 50 μm. **B**. Typical example of Masson's trichrome staining = 50 μm.

ONP-302 treatment was associated with a marked reduction in αSMA staining in ONP-302 treated mice when compared to control group **(Figure 6A).** This result was consistent with reduced *αSma* expression and increased frequency of apoptotic CAFs in the TME after treatment with ONP-302. Masson's trichrome staining allows for visualization of tissue composition and detection of collagen fibers. The collagen fibers stain blue and the nuclei stains black, with a red background. To capture changes in tumors caused by ONP-302 before substantial antitumor effect was visible, mice were treated with ONP-302 for only two weeks. By that time, ONP-302 caused modest decrease in tumor growth (**Figure S6**). In control mice, tumors showed homogeneous staining of tumor parenchyma. In striking contrast, in ONP-302 treated mice, tumors had large areas of necrosis (**Figure 6B**) supporting tumor tissue destruction by ONP-302 mediated effects.

## Discussion

**Figure 7:**
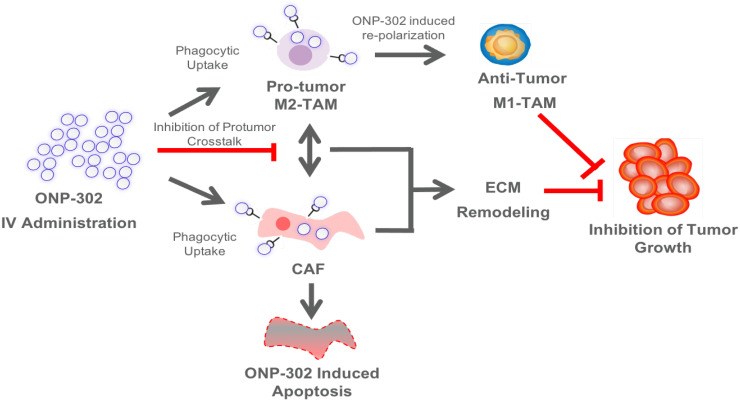
Schematic Illustration of the ONP-302 Mechanism. Details are provided in the text.

ONP-302 demonstrated strong efficacy in three different tumor models of lung cancer, melanoma, and colon cancer with distinct growth kinetics and levels of immune infiltrate. We examined several potential mechanisms of actions of ONP-302 efficacy. ONP-302 did not appear to have inherent tumoricidal properties as in vitro incubation of tumor cells with microgram concentrations of the particles did not induce cell-death. Our original hypotheses postulated that ONP-302 would inhibit tumor growth by altering the composition of pro-tumorigenic TAMs and MDSCs in the TME. MDSCs and TME engage in immune suppressive and pro-tumorigenic activities, which promote tumor progression and enable tumor immune evasion 43. We expected ONP- 302 targeting of MDSCs and TAMs infiltration in the TME to disrupt the immune suppressive and pro-tumor signaling pathways in the TME subsequently unleashing the anti-tumor effector immune response leading to effective tumor growth control. We observed evidence of disruptions in immune-suppressive and pro-tumor signaling pathways in the TME, not due to reduction in the total numbers of MDSCs and TAMs, but due to changes in TAMs and CAFs which are major cell types implicated in tumor promotion. ONP-302 induced a shift in TAM gene expression from the pro-tumor M2-like to the pro-inflammatory M1-like pattern. ONP-302 treatment caused apoptosis of CAFs and altered the functionality of remaining cells by inhibiting the expression of genes associated with pro-tumorigenic functions such as ECM remodeling (**Figure 7**).

To date, nanoparticle-based approaches for the treatment of cancers have been limited to the use of nanoparticles as drug carriers. Research and development of these nanoparticle drug carriers has primarily focused on engineering the physiochemical properties of nanoparticles for improved bioavailability, active and passive targeting for drug delivery to specific cell types and tissues, and reducing the toxicity of the drugs being delivered. The clinical translation of these approaches has been limited by manufacturing challenges, off-target drug delivery, limited efficacy due to reliance on drug-dependent effects on singular pathways in a disease state involving interplay between multiple signaling pathways, unwanted interactions with the immune system, and risks of toxicities (44-47). To our knowledge, this is first demonstration that biodegradable nanoparticles, free from other drugs or bioactive agents, can be designed to be inherently immunomodulatory having pleiotropic effects on multiple pro-tumor signaling pathways which can be leveraged for the safe and effective treatment of cancers. The physiochemical properties of ONP-302 are designed to overcome challenges encountered by traditional nanoparticle technologies as it does not rely on immunomodulatory drug delivery at the tumor site. The physiochemical properties of ONP-302 target the particles for uptake by phagocytic cells such as monocytes, neutrophils, and macrophages 31. The resulting immunomodulatory effect on these cell-types was shown to be dependent on scavenger receptor MARCO 31. In line with previous observations, we found MDSCs and TAMs in the TME were ONP-302-positive. It remains to be determined whether the MDSC precursors or the TAMs (or both) take up the infused ONP-302 particles in the periphery or at the tumor site. Additionally, ONP-302 uptake by non-immune cell-types had not been explored previously. We found ONP- 302-positive cancer-associated fibroblasts in the TME. Minimal ONP-302 uptake was observed in normal fibroblast outside the TME (e.g spleen) indicating ONP-302 may interfere with MARCO-expressing CAFs engaged in pro-tumorigenic activity and ECM remodeling in the TME. In the tumor stroma, the interplay of TAMs and MDSCs with CAFs promotes tumor growth and progression via chemokine and growth-factor signaling pathways 48,49. Myeloid-derived cells, such as MDSCs and TAMs, are found in close vicinity of CAFs in the TME and are known to interact via complex paracrine signaling pathways involving chemokines, cytokines, and growth factors 40,41. Our data indicate that gene expression alterations in TAMs and CAFs after ONP- 302 treatment may disrupt the pro-tumorigenic interplay between these cell-types in the TME. For example, ONP-302 treatment led to a marked reduction in Cxcl1 expression by CAFs that promote PMN-MDSC infiltration into the TME in line with our observation of reduced PMN-MDSCs in the LLC TME after ONP-302 treatment 50. ONP-302 treatment inhibited Fap expression, which would potentially interfere with FAP-mediated remodeling of the ECM (**Figure 7**). Disruption of the ECM remodeling would modulate scavenger receptor MARCO and SR-A dependent macrophage cell- adhesion and migration into the TME 51. Importantly, increased levels of MARCO-positive myeloid cells within the TME predict tumor progression and poor outcomes 52. Consistent with the known roles of TAMs and CAFs in promoting tumor growth and our observation of the ability of ONP-302 to disrupt signaling pathways and functions associated with these roles, treatment with ONP-302 resulted in slower tumor growth 48,49. The finding that ONP-302 treatment could affect both myeloid-derived and stromal cells in the TME is noteworthy. CAF were also implicated in regulation of angiogenesis, which in turn promote tumor progression 53,54. In addition, shift macrophage polarization towards M1 also can reduce angiogenesis 55-57. Thus ONP-302 mechanism appears to be highly effective at slowing tumor growth, with advantages over previously failed therapeutic approaches that targeted myeloid cells and CAFs in isolation. These pathways, while implicated by our data, need to be further examined in future studies.

Based on our initial hypotheses, we expected disruptions in immune suppressive and pro-tumor signaling pathways to result in the activation of T cell-mediated tumor killing. ONP-302 was able to decrease PMN-MDSC suppressive activity. However, ONP-302 efficacy was evident even after CD8+ T cell depletion via anti-CD8 monoclonal antibody treatment and in immune compromised mice. These results suggest that the reduced tumor burden observed after ONP-302 treatment was not entirely driven by activated T cell-mediated tumor killing. In addition to T cells, NK cells are also known to play an important role in mounting an anti-tumor immune response. We observed a statistically significant increase in NK cells in the TME after ONP-302 treatment. The role of NK cells in mediating the anti-tumor effects of ONP-302 needs to be explored further and is subject of further investigation by our group. At present, our data indicate that the slowing of tumor growth after ONP-302 treatment is due to disruptions in known signaling pathways involving TAMs and CAFs, pathways typically supporting tumor growth. These data taken in concert indicate the activity of ONP-302 is pleotropic and affects multiple pathways.

## Supplementary Material

Supplementary figures and tables.
