# Supplementary material for: ONP-302 Nanoparticles Inhibit Tumor Growth By Altering Tumor-Associated Macrophages And Cancer-Associated Fibroblasts
Source: J Cancer. 2022 Mar 28;13(6):1933–44. doi: 10.7150/jca.69338 (PMC8990435; doi:10.7150/jca.69338)
Supplement: Supplementary file 1 — Supplementary figures and tables. [file jcav13p1933s1.pdf]

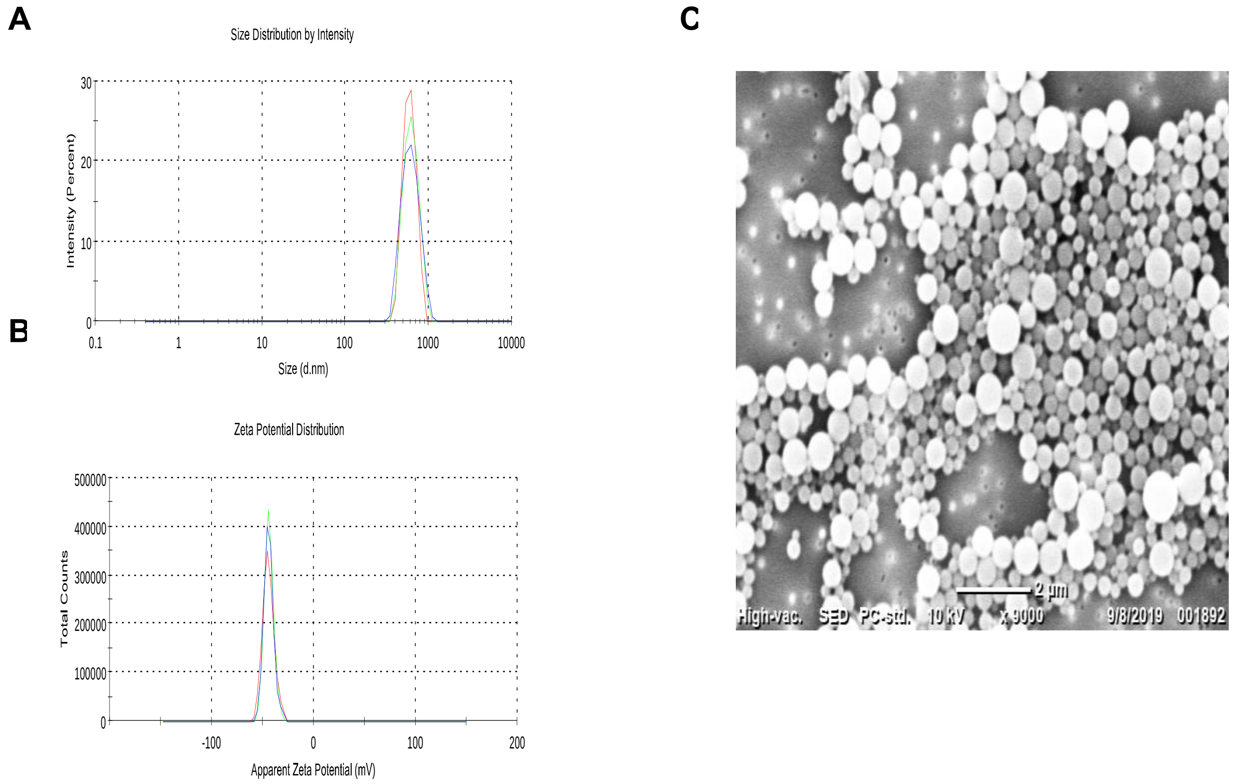

Figure S1. Physiochemical characterization of ONP-302 nanoparticles. Size **(A)** and zeta potential **(B)** of ONP-302 were determined using Dynamic Light Scattering (DLS). Shown are DLS plots for size and zeta potential from three measurements. **C.** Particle size and morphology was examined using a Scanning Electron Microscope. Shown is a representative image of ONP-302 particles at a 5000x magnification.

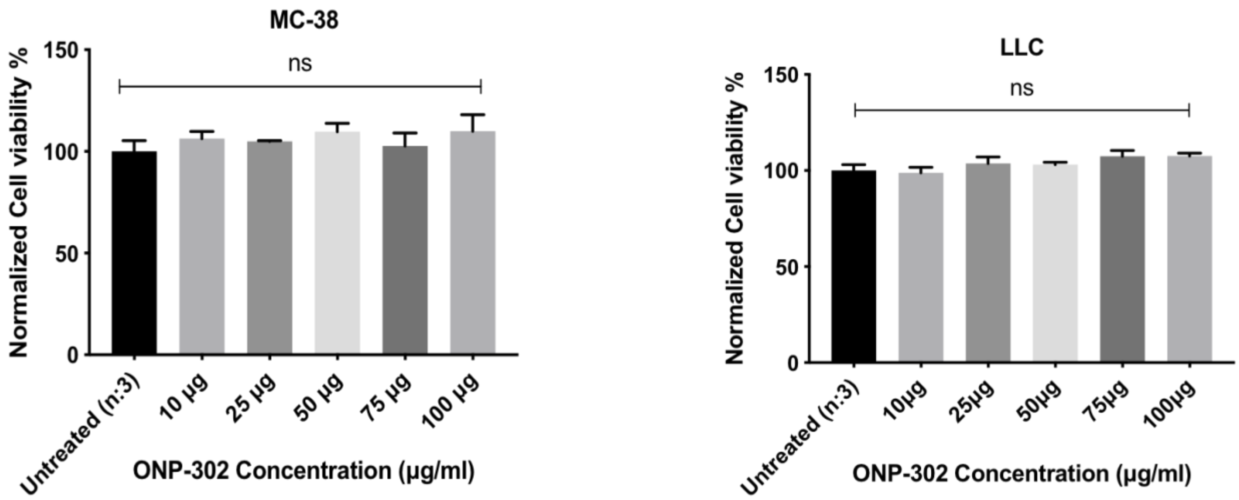

Figure S2: Effect of ONP-302 on tumor cell-viability in vitro. MC-38 and LLC tumor cells incubated in vitro with indicated concentrations of ONP-302. Cell-viability was assessed using resazurin dye. Shown is absorbance indicative of cell-viability in **(A)** MC-38 and **(B)** LLC tumor cells incubated with indicated concentrations of ONP-302. N=4.

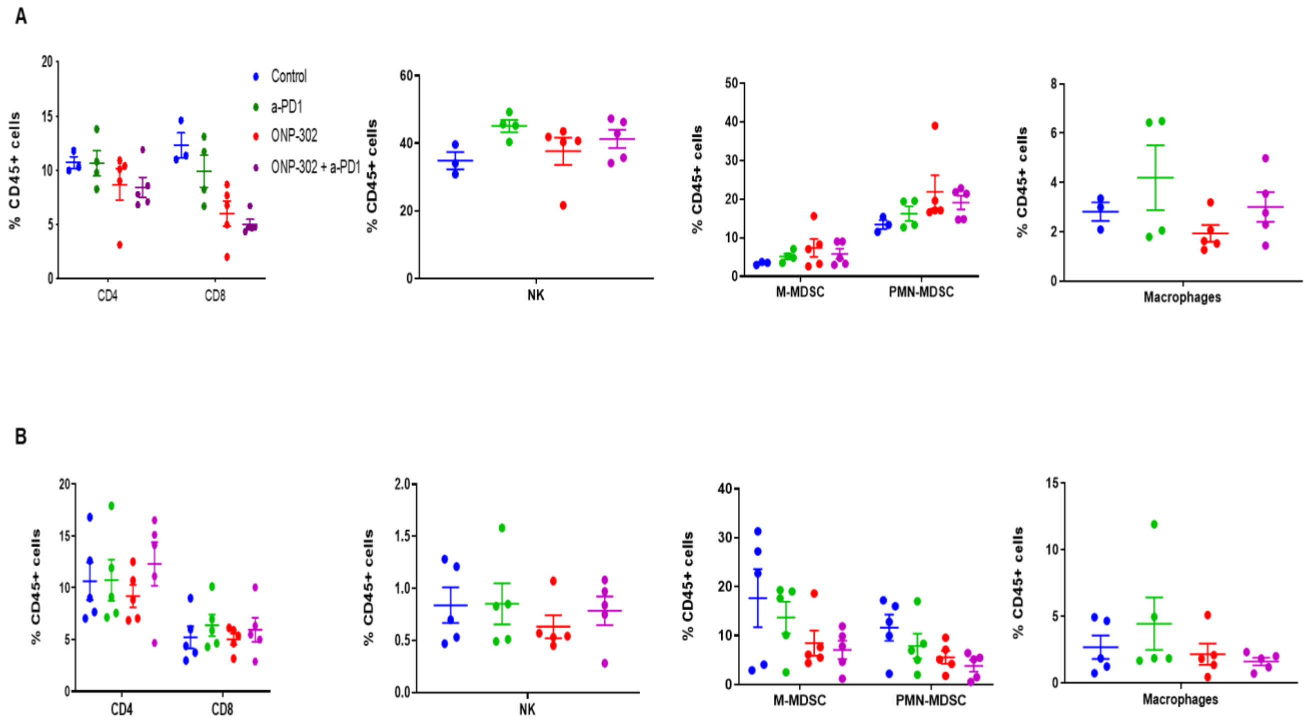

Figure S3: Effect of ONP-302 on immune-cell composition in the spleen of tumor-bearing mice. LLC and MC-38 tumor-bearing C57BL/6 mice were treated with vehicle or ONP-302 50mg/kg intravenously twice per week for 2 weeks. Single-cell suspensions were prepared from the spleen and immune cell composition was analyzed by flow cytometry. **A.** Proportion of T-cells ($CD3^{+}CD4^{+}$ or $CD3^{+}CD8^{+}$), NK cells ($CD3^{-}$ $NK1.1^{+}$), monocytes ($CD11b^{+}$ $Ly6C^{hi}$ $Ly6G^{-}$), neutrophils ($CD11b^{+}$ $Ly6C^{-}$ $Ly6G^{+}$), and macrophages ($CD11b^{+}F4/80^{+}$) in the spleens of LLC tumor-bearing mice. **B.** Proportion of T-cells ($CD3^{+}CD4^{+}$ or $CD3^{+}CD8^{+}$), NK cells ($CD3^{-}$ $NK1.1^{+}$), monocytes ($CD11b^{+}$ $Ly6C^{hi}$ $Ly6G^{-}$), neutrophils ($CD11b^{+}$ $Ly6C^{-}$ $Ly6G^{+}$), and macrophages in ($CD11b^{+}F4/80^{+}$) in the spleens of MC-38 tumor-bearing mice.

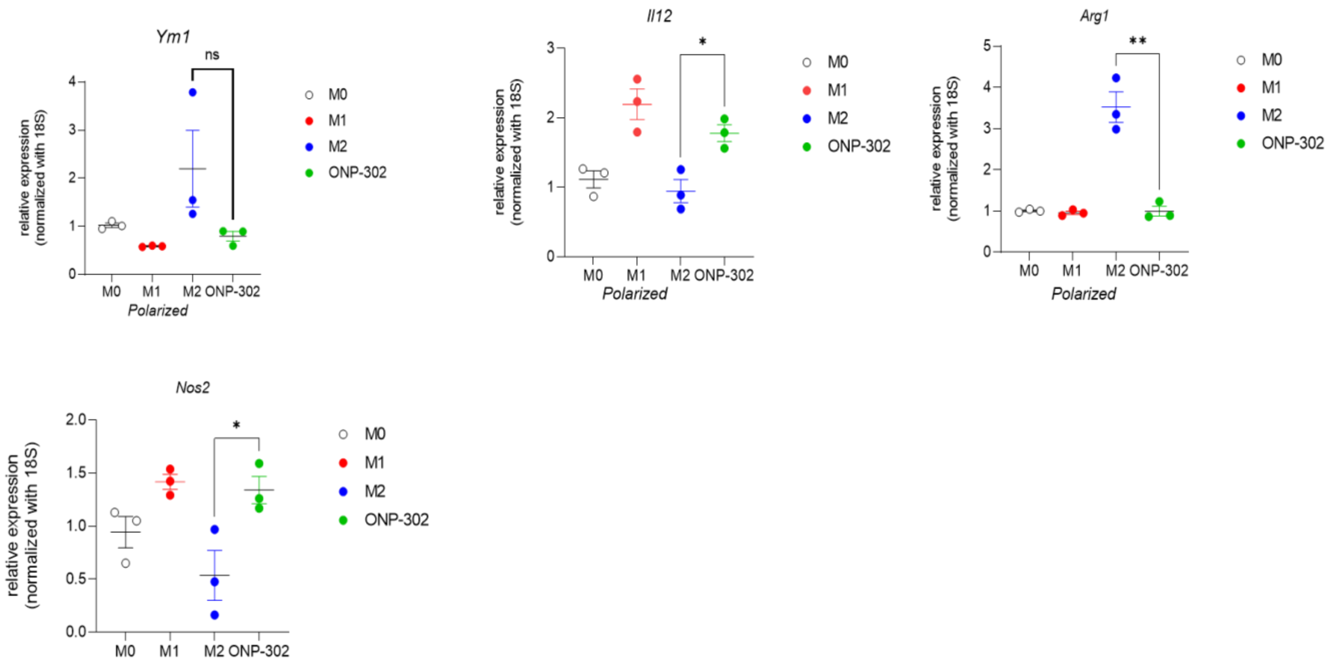

Figure S4. Effect of ONP-302 on macrophage polarization. Macrophages were generated from bone marrow monocytes by 5 days culture with murine M-CSF (10 ng/mL). They were polarized to M1 or M2 type of cells by 24 hr incubation with LPS (10ng/mL) /mIFN-γ (10ng/mL) or mIL-4 (10ng/mL)/mIL-13(10ng/mL) respectively. In parallel macrophages were cultured with ONP-302. Expression of indicated genes was evaluated. P values were calculated by one-way ANOVA test with correction for multiple comparisons. * -p<0.05; **-p<0.01

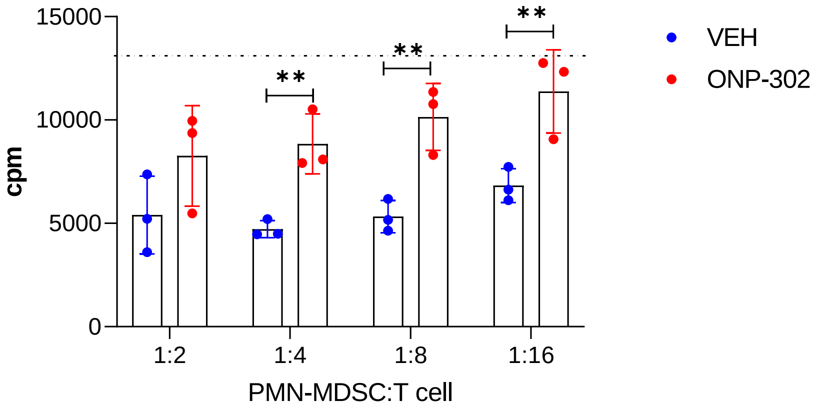

Figure S5. PMN-MDSC suppressive activity after treatment with ONP-302. Single cell suspension of Vehicle (saline) and ONP-302 treated tumors were prepared (Miltenyi biotec) and PMN-MDSCs (CD11b$^+$Ly6G$^+$Ly6C$^-$) were sorted on BD FACs Aria II cell sorter. Sorted PMN-MDSCs were plated in triplicates in U-bottom 96 well cell culture plates in RPMI-1640 with 10% FBS (ThermoFisher) and β-mercaptoethanol (1:1000, SIGMA). OT-1 splenocytes were added at different ratios together with SIINFEKL peptides (0.1 ng/ml, SIGMA) to induce antigen-specific T cell proliferation. After 48hr incubation 1 µCu [^{3}H] thymidine (PerkinElmer) was added, and cells were incubated for additional 18 hr and radioactivity was measured on Microbeta 2 microplate counter, PerkinElmer. Incorporation of [^{3}H] thymidine in proliferating T cells was measured and is represented in counts per minute (cpm). N=3. P values were calculated in unpaired, two-sided, Student's t-test. *-p<0.05; **-p<0.01.

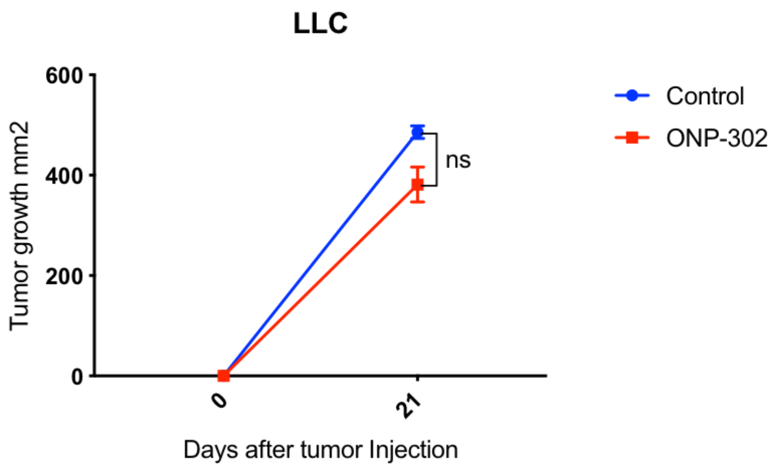

Figure S6. Effect of ONP-302 on LLC : C57BL/6 mice (n=5) were injected with 0.5 x 10^6 LLC tumor cells. Treatments with ONP-302 intravenously 50 mg/kg biw started at ~50 mm^2 tumor size for one and half weeks (3 doses) after tumor injection. Mice were euthanized and tumors were collected before the significant tumor reduction for the immunohistochemistry (IHC) to evaluate the apoptotic Cancer Associated Fibroblasts (CAF).

Supplementary Table 1: Characteristics of nanoparticles

Nanoparticle	Size (nm)	PDI	Zeta Potential
ONP-302	568 ± 13	0.21	-41.5 ± 0.42
ONP-302 (OVA-Alexa-Fluor 647)	541 ± 14.7	0.18	-41.9 ± 0.03

Supplementary Table 2: Antibodies used in the study

**Antibody**	**Source**	**Catalogue Number**
Rat anti-mouse CD140a	BD Biosciences	Cat#562776
Rat anti-mouse CD326	BD Biosciences	Cat#563478
Rat Anti-Mouse CD45	BD Biosciences	Cat# 553080
Rat anti-Mouse F4/80	BD Biosciences	Cat#565411
Rat anti- Mouse CD11b	BD Biosciences	Cat#557960
Rat Anti-Mouse Ly-6G	BD Biosciences	Cat#551461
Rat Anti-Mouse Ly6C	BD Biosciences	Cat#560593
Rat Anti-Mouse CD8a	BD Biosciences	Cat#553031
Hamster Anti-Mouse CD3	BD Biosciences	Cat#552774
Rat anti-mouse CD4	BioLegend	Cat#100437
InVivoMab anti-mouse PD-1	Bioxcell	Clone#RMP1-14
InVivoMab anti-mouse CD8a	Bioxcell	Cat# BE0004-1-A0
Annexin V (FITC)	BD Biosciences	Cat# 556419
Anti-actin-αSmooth-Muscle	Sigma	Cat#A5228
Rabbit anti-mouse IgG AF-488	Invitrogen	Cat# A27023.

Supplementary Table 3: Primers used in the study

Gene	Sequence	Sequence ID
Arginase-1	5’ AGGAACTGGCTGAAGTGGTTA 3'<br>5’ GATGAGAAAGGAAAGTGGCTGT 3’	NM_007482.3
CD206	5’CAGGTGTGGGCTCAGGTAGT 3’<br>5’TGTGGTGAGCTGAAAGGTGA 3’	NM_008625.2
Ym-1	5’GGGCATACCTTTATCCTGAG 3’<br>5’CCACTGAAGTCATCCATGTC 3’	NM_009892.3
iNOS (NOS2)	5’CAGAGGACCCAGAGACAAGC 3'<br>5’TGCTGAAACATTTCCTGTGC 3’	NM_001313921.1
α-SMA	5’ATCATTGCCCCTCCAGAACG 3’<br>5’GCTAGGCCAGGGCTACAAGT 3’	NM_007392.3
Vimentin	5’CGCTCCTACGATTCACAGCC 3’<br>5’TGTGGACGTGGTCACATAGC 3’	NM_011701.4
FAP	5’CCAGGCGATGTGGTACTCTG 3’<br>5’CTAACCTCCTGAGCCCTCCTA 3’	NM_007986.3
Col1A1	5’CTTCCTGCCCACTTGGCTTA 3’<br>5’GGGTGCTGGGTAGGGAAGTA 3’	NM_007742.4
IFN-γ	5’CAAGACTGTGATTGCGGGGT 3’<br>5’AGCCAAGACTGTGATTGCGG 3’	NM_008337.4
IL-12a	5’GTCTACACTGCTGCTGAAATCTT 3’<br>5’GCCAAAAAGAGGAGGTAGCG 3’	NM_001159424.2
GAPDH	5’GTTGTCTCCTGCGACTTCA 3’<br>5’GGTGGTCCAGGGTTTCTTA 3’	NM_001289726.1
